# Optimal indication criteria for neoadjuvant chemotherapy in patients with resectable colorectal liver metastases

**DOI:** 10.1186/s12957-019-1641-5

**Published:** 2019-06-13

**Authors:** Hirofumi Ichida, Yoshihiro Mise, Hiromichi Ito, Takeaki Ishizawa, Yosuke Inoue, Yu Takahashi, Eiji Shinozaki, Kensei Yamaguchi, Akio Saiura

**Affiliations:** 1 Department of Gastroenterological Surgery, Cancer Institute Hospital, Japanese Foundation for Cancer Research, 3-8-31 Ariake, Koto-ku, Tokyo, 135-8550 Japan; 2 Department of Gastroenterology, Cancer Institute Hospital, Japanese Foundation for Cancer Research, 3-8-31 Ariake, Koto-ku, Tokyo, 135-8550 Japan

**Keywords:** Colorectal cancer, Liver metastases, Chemotherapy, Liver resection

## Abstract

**Background:**

There are no optimal indication criteria for neoadjuvant chemotherapy (NAC) in patients with resectable colorectal liver metastases (CLM). The aim of this study was to prospectively assess the survival benefit of selective NAC administration in this patient population based on tumor characteristics.

**Methods:**

Borderline resectable CLM (BR-CLM) were defined as four or more liver metastases, CLM larger than 5 cm, or CLM with concomitant resectable extrahepatic metastases. From 2010 to 2015, NAC was administered to BR-CLM patients. Upfront surgery without NAC was performed to patients having clearly resectable CLM (less than 3 lesions, smaller than 5 cm, and no extrahepatic metastases: CR-US group). Survival outcomes of the two groups were assessed.

**Results:**

The BR-NAC group comprised 73 patients and the CR-US group 172. All patients in the BR-NAC group underwent subsequent resection, as none showed disease progression or chemotherapy-associated liver damage. The 3- and 5-year overall survival rates of the CR-US group were 83.0% and 74.0%, while patients in the BR-NAC group had comparable 3-year and 5-year overall survivals (80.5% and 66.6%, *P* = 0.397).

**Conclusion:**

Defining BR-CLM based on tumor characteristics optimizes patient selection for NAC. Favorable overall survival can be achieved by upfront surgery in patients with clearly resectable CLM and by NAC in patients with BR-CLM.

## Background

Modern chemotherapy, which includes oxaliplatin or irinotecan combined with targeted therapy, has dramatically changed the treatment strategy for colorectal liver metastases (CLM). The prognoses of patients receiving palliative chemotherapy for unresectable CLM have improved, with the median survival time now 30 months [1]. Additionally, effective chemotherapy in selected patients can produce resectable tumors from what were initially unresectable tumors [2, 3].

However, the clinical benefit of neoadjuvant chemotherapy (NAC) has yet to be established in patients with resectable CLM. The European Organization for Research and Treatment of Cancer (EORTC) trial 40983 reported that perioperative chemotherapy prolonged recurrence-free survival (RFS) [4, 5]. However, no prospective studies have demonstrated improved overall survival (OS) in patients receiving NAC for resectable CLM. Although the theoretical benefits of controlling the disease prior to surgery are well accepted, optimizing patient selection for NAC is a critical issue because preoperative chemotherapy can deprive some patients with resectable tumors of access to surgery due to the development of chemotherapy-related liver damage or disease progression during NAC [6–9].

Several recent retrospective analyses have suggested that selective NAC for patients with high-risk profiles is associated with improved OS after surgery for resectable CLM [10–12]. Latest ESMO consensus guidelines allow upfront surgery without NAC in patients having technically “easy” and oncologically “excellent” CLM [1]. However, no practical criteria have been set regarding whether to administer NAC or not in patients with resectable CLM.

We define borderline resectable CLM (BR-CLM) as more than four metastases, metastases larger than 5 cm, or concomitant resectable extrahepatic disease (EHD). Other resectable CLM are defined as clearly resectable CLM (CR-CLM). We administered NAC for patients with BR-CLM, and upfront surgery without NAC was performed in patients with CR-CLM. The aim of this study was to assess the prognostic validity of our indication criteria for NAC in patients with resectable CLM.

## Methods

### Study cohort

Beginning in January 2010, NAC was administered to patients who had BR-CLM (as defined below) at the Cancer Institute Hospital of Japanese Foundation for Cancer Research (Tokyo, Japan). This study was approved by the Institutional Review Board.

Survival outcomes were compared between BR-CLM patients who received NAC followed by surgery (BR-NAC group) and those who had CR-CLM with a low-risk profile and underwent upfront surgery without NAC (CR-US group). Recurrence patterns of the CR-US and BR-NAC groups were compared with those of a historical control group of patients who received upfront surgery for BR-CLM without NAC (BR-US group) from January 2005 to December 2009. Patients who underwent upfront surgery for BR-CLM after 2010 were also included in the BR-US group.

The exclusion criteria were as follows: (1) CLM during adjuvant chemotherapy for the primary tumor, (2) unspecified preoperative chemotherapy at a previous hospital, (3) death from another disease within 1 year of surger, (4) R1/2 resection during primary tumor resection, and (5) recurrent CLM.

### Definition of resectability

**Fig. 1:**
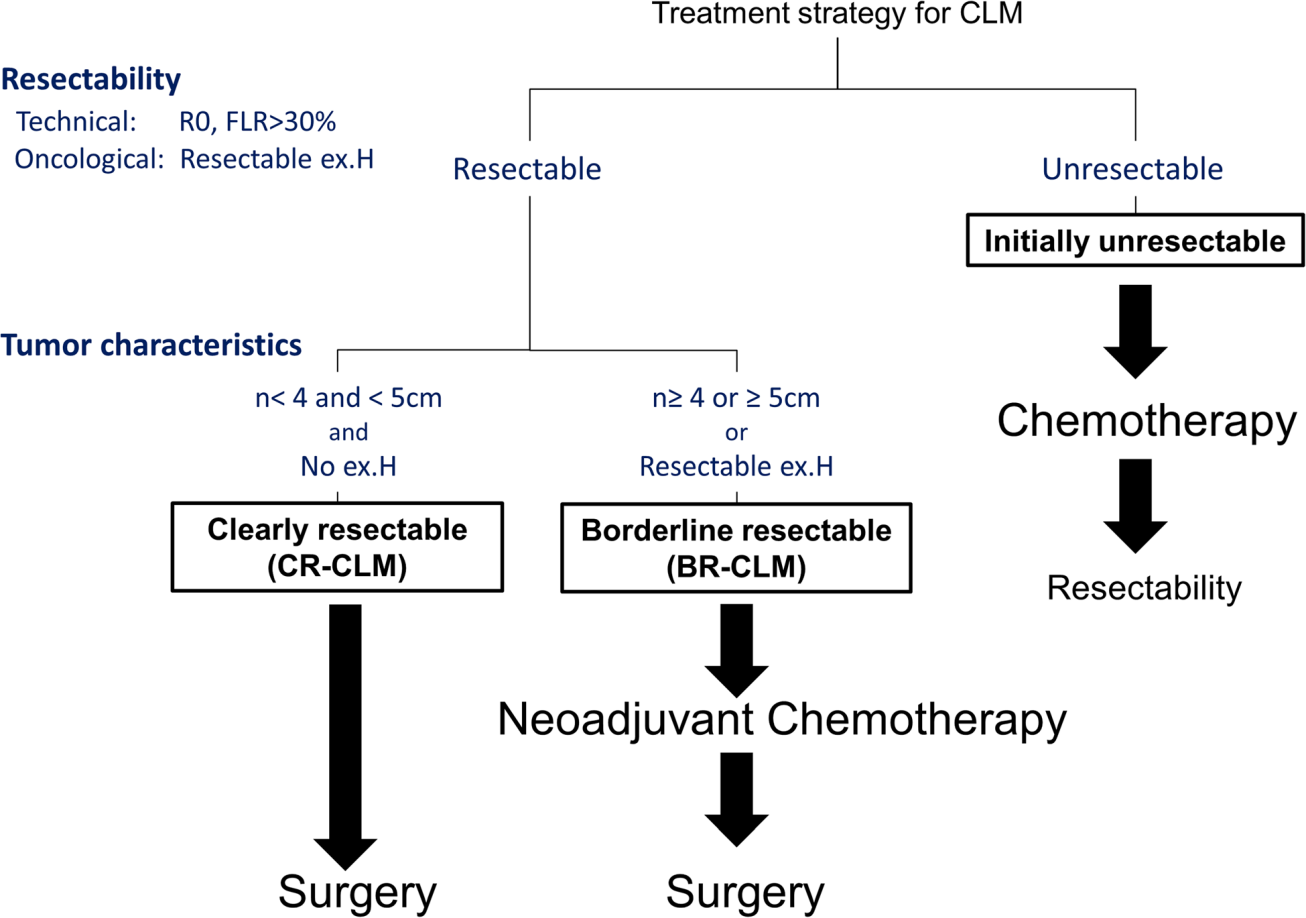
Definition of resectability and the treatment strategies for CLM

Figure 1 summarizes our definition of resectability and our CLM treatment strategies. First, CLM resectability was established by reviewing computed tomography and magnetic resonance imaging obtained at the initial visit from technical and oncological viewpoints: technically resectable CLM were defined as tumors that could be removed with clear margins leaving greater than 30% of residual liver parenchymal volume regardless of tumor number or size, including resectable EHD, whereas oncologically unresectable CLM were defined as those with concomitant unresectable EHD. Patients with resectable CLM were then divided into two groups according to tumor characteristics: the CR-CLM group, which had fewer than four tumors all less than 5 cm in diameter and no EHD, and the BR-CLM group, which had four or more tumors, a tumor diameter greater than or equal to 5 cm, or concomitant resectable EHD. These factors for classification of the groups were based on the analysis of the data of surgical outcomes for CLM before 2009 in our institute and previous reports that analyzed high-risk factors for postoperative RFS [13–15].

### Treatment strategies for CR-CLM and BR-CLM

Six cycles of oxaliplatin-based NAC were administered to selected BR-CLM patients following the approval of a multidisciplinary team conference [16], except for patients who were included in two randomized controlled studies [17, 18]. Upfront surgery without NAC was the standard approach for CR-CLM throughout this study.

Surgery was performed 4 weeks after the last cycle of oxaliplatin-based chemotherapy or 6 weeks after the last bevacizumab cycle [19, 20]. Provided that R0 resection was deemed possible, surgery was performed regardless of the NAC response. Details of the surgical procedures have been described elsewhere [21]. Radiofrequency ablation was not used in this study. Synchronous metastasis was defined by disease-free interval shorter than 6 months. Postoperative complications were classified according to the Clavien–Dindo classification [22], with grade 3a or worse defined as a major complication. All complications that developed within 90 days after surgery were included [23]. R0 resection was defined as no microscopic evidence of tumor in the resection margin, regardless of the existent of concomitant resectable lung metastases. Patients were followed up every 3 months during the first 2 years and every 6 months thereafter. Recurrent CLM were treated the same as the initial CLM.

### Long-term outcomes

OS and RFS were defined as the interval from the date of starting chemotherapy in the BR-NAC group or the date of hepatic resection in the CR-US group to the date of death (OS) or recurrence (RFS). To investigate the impact of NAC on recurrence in patients with BR-CLM, the recurrence patterns of the BR-NAC and CR-US groups were compared with those of the historical control BR-CLM patients who underwent upfront surgery between 2005 and 2015 (BR-US group).

### Statistical analysis

Categorical variables were compared using the *χ*^2^ test, and continuous variables by the independent sample *t* test. Survival curves were calculated by the Kaplan–Meier method and compared by the log-rank test. In the CR-US group, univariate and multivariate analyses were performed to assess prognostic factors affecting survival. A Cox proportional hazards model was adopted for the multivariate analyses. Background elimination methods with a *P* value < 0.1 for variable elimination were used to select the final model. *P* < 0.05 was considered statistically significant. Statistical analyses were performed using JMP v12.2 (SAS Institute, Cary, NC, USA).

## Results

### Study subjects

**Fig. 2:**
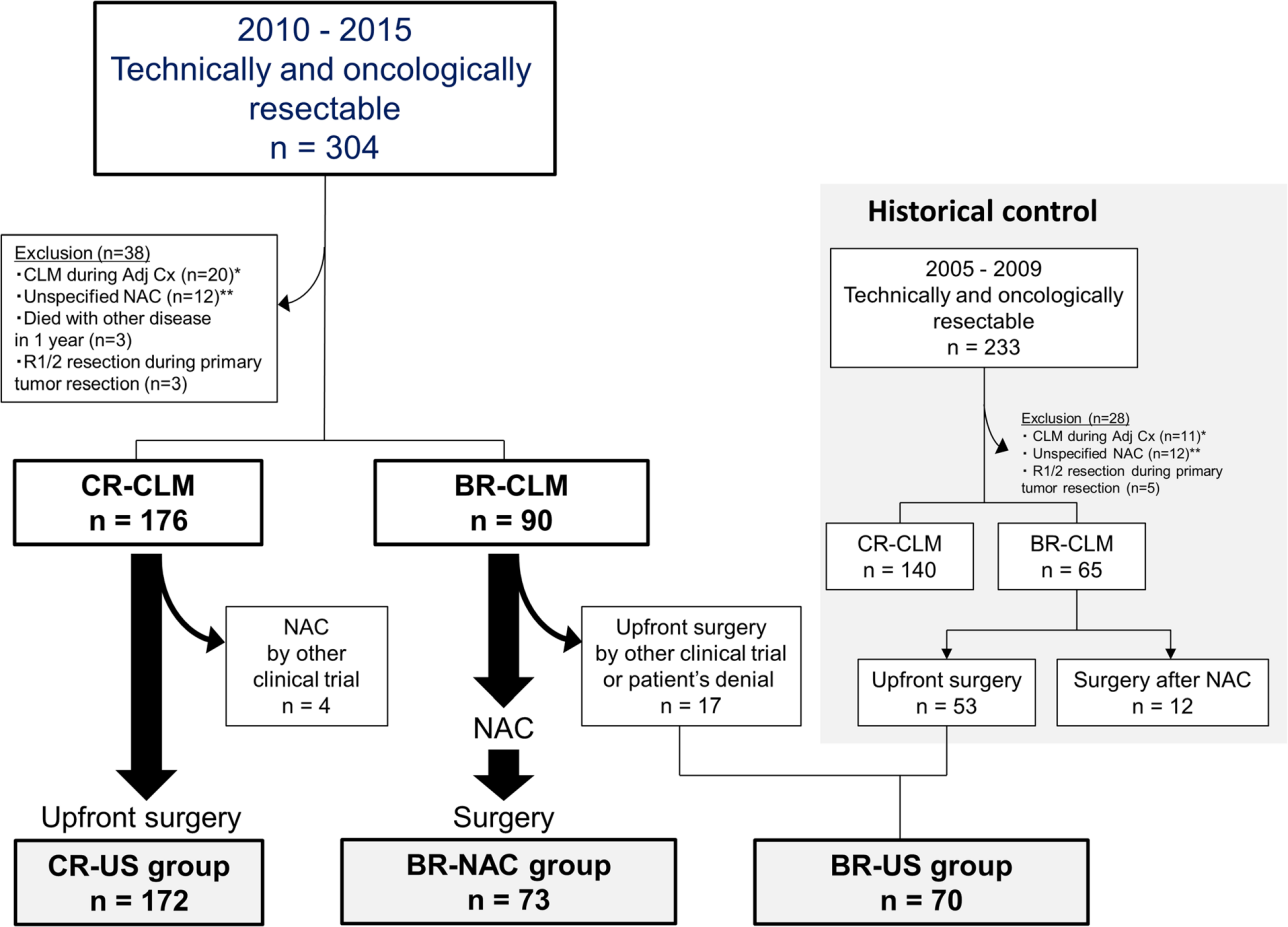
Selection process for the CR-US, BR-NAC, and BR-US groups. *CLM during adjuvant chemotherapy of the primary tumor. **Unspecified preoperative chemotherapy at previous hospital

Between January 2010 and December 2015, 382 consecutive patients underwent hepatectomy for CLM. Among them, 78 had initially unresectable CLM, while the remaining 304 were judged to have technically and oncologically resectable disease at their initial visit. After excluding 38 patients who met the exclusion criteria, there were 90 BR-CLM and 176 CR-CLM patients. Seventeen patients underwent upfront surgery for BR-CLM because they participated in other clinical trials (*n* = 5) [18] or refused to be included in this study (*n* = 12). In total, 73 patients comprised the BR-NAC group. Among the CR-CLM patients, four received NAC prior to surgery because they participated in other clinical trials; thus, the remaining 172 patients comprised the CR-US group. From January 2005 to December 2009, 65 patients underwent hepatectomy for BR-CLM. After excluding 12 patients who received NAC at other hospitals, there were 53 remaining patients from this cohort and 17 patients who underwent upfront surgery for BR-CLM after 2010 (together the BR-US group). Figure 2 summarizes the selection process, and Table 1 and Table 2 show baseline characteristics and surgical outcomes of the BR-NAC and CR-US, respectively.

**Table 1:**
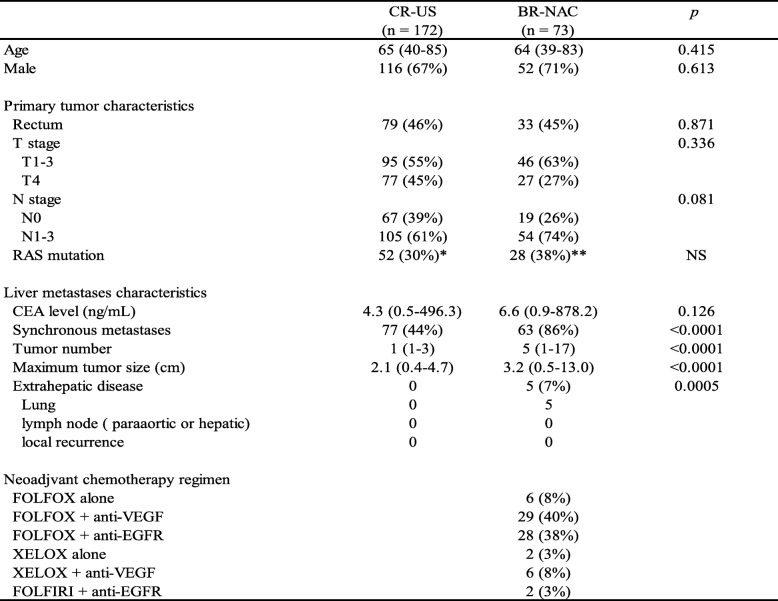
Patient characteristics

*Not available for 105 patients

**Not available for 30 patients

**Table 2:**
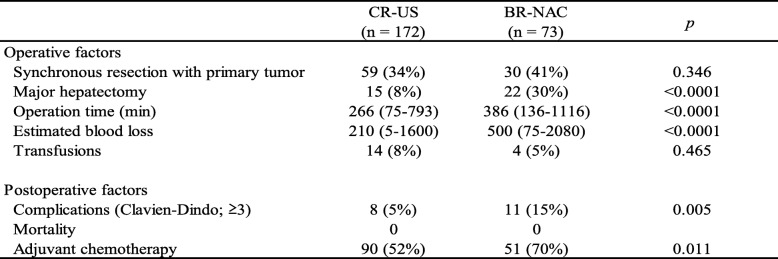
Surgical outcomes

All of the patients in these groups received R0 hepatic resection. Although single paraaortic or hepatic lymph node metastasis was defined to be resectable EHD, all of the concomitant EHDs in the BR-NAC group were only lung metastases through the study period.

### NAC responses

No patients who received NAC abandoned the subsequent hepatic resection due to disease progression. The most commonly used NAC regimens were FOLFOX (63 patients, 86%), followed by XELOX (eight patients, 11%), and FOLFIRI (two patients, 3%) (Table 1). The patients who received FOLFIRI did so because of numbness in their hands, a possible adverse effect of oxaliplatin, which may have affected their subsequent ability to work.

According to the RECIST 1.1 criteria [24], 49 patients (67%) achieved partial responses (PR), there were no (0%) complete responses (CR), 14 (19%) showed stable disease (SD), and 10 (14%) showed disease progression (PD).

### Long-term survival

**Fig. 3:**
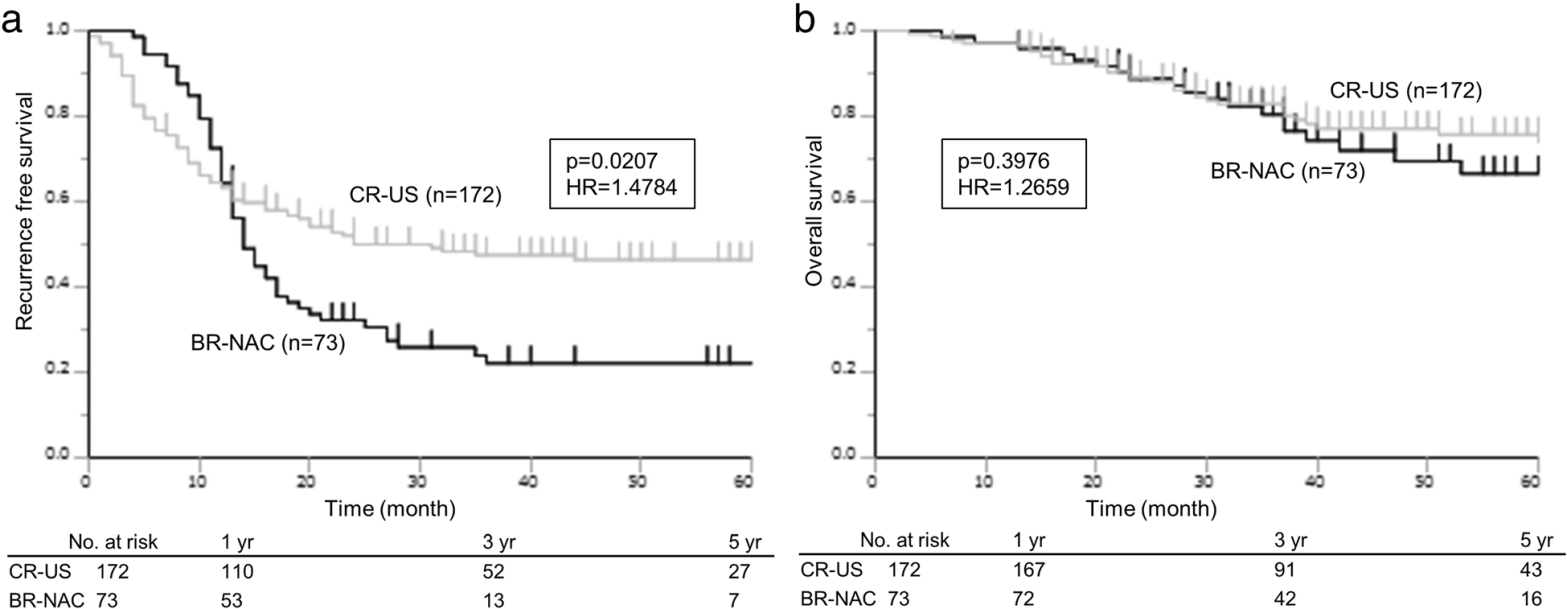
Long-term survival in the BR-NAC and CR-US groups. **a** Recurrence-free survival. **b** Overall survival. Survival curves were calculated from the date of chemotherapy initiation in the BR-NAC group and from the date of hepatic resection in the CR-US group

The median follow-up periods in the BR-NAC and CR-US groups were 37 (range 6–83) months and 37 (range 3–83) months, respectively (*P* = 0.5834). The 1-, 3-, and 5-year OS rates in the BR-NAC and CR-US groups were 97.3%, 80.5%, and 66.6%, respectively, versus 97.1%, 83.0%, and 74.0%, respectively (*P* = 0.3976). As shown in Fig. 3, the 1-, 3-, and 5-year RFS rates in the BR-NAC group (64.4%, 22.1%, and 22.1%, respectively) were significantly shorter than in the CR-US group (63.3%, 47.6%, and 46.5%, respectively; *P* = 0.0207).

In the CR-US group, multivariate analysis revealed that disease-free interval (DFI) from primary disease was the only predictive factor of impaired OS (hazard ratio 3.149, *P* = 0.036, Table 3).

**Table 3:**
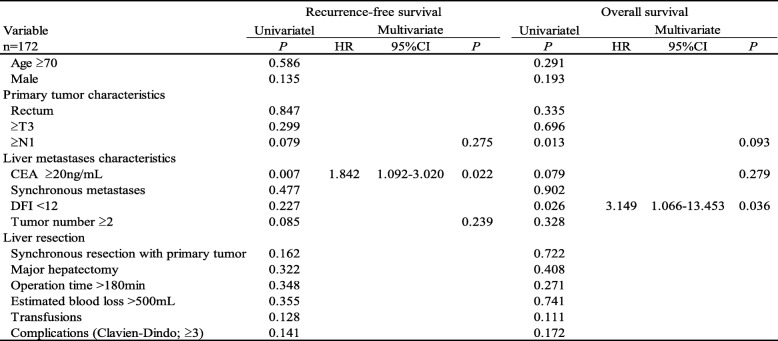
Univariate and multivariate analysis of clinicopathological variables in CR-US associated with RFS and OS

### Recurrence patterns after initial hepatic resection

**Fig. 4:**
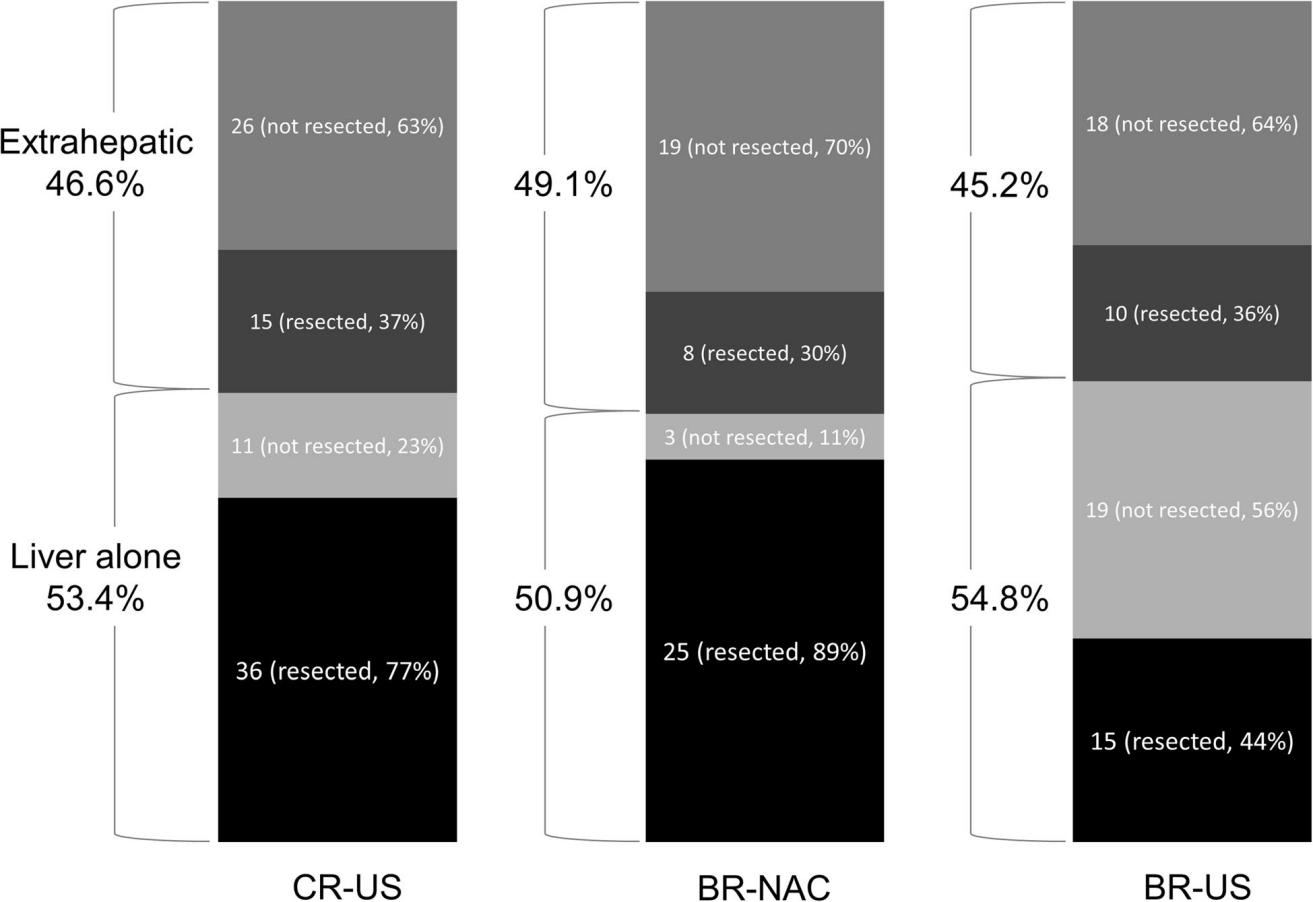
Patterns of recurrence after hepatic resection

In total, 88 (51.2%) patients in the CR-US group, 55 (75.3%) in the BR-NAC group, and 62 (88.6%) in the BR-US group developed recurrence after initial hepatic resection (BR-NAC vs CR-US, *P* = 0.0004; BR-NAC vs BR-US, *P* = 0.0403). The incidence of liver recurrence in the BR-NAC group (50.9%) was not statistically different from that of the CR-US (53.4%; *P* = 0.7709) and BR-US (54.8%; *P* = 0.6708) groups. However, the rate of re-hepatectomy for recurrences in the BR-NAC group (89.3%) was similar to that of the CR-US group (76.6%; *P* = 0.1725) and was better than that of the BR-US group (44.1%; *P* = 0.0002) (Fig. 4).

## Discussion

This is the study to define resectable CLM with high-risk profiles as BR-CLM based on tumor characteristics and selectively administer NAC to BR-CLM patients. This study demonstrated that upfront surgery for CR-CLM, which has low-risk profiles, offered a favorable 5-year OS rate of 74.0%, while patients in the BR-NAC group had comparable 5-year OS rates (66.6%) to the CR-US group. Analysis of recurrence patterns revealed that NAC for BR-CLM reduced unresectable liver recurrences after hepatectomy, which may have led to the favorable OS rates in the BR-NAC group. Additionally, selective NAC for BR-CLM was feasible, as no patients in the BR-NAC group were excluded from subsequent resection due to disease progression or chemotherapy-associated liver damage.

In this study, upfront surgery without NAC in CR-CLM patients achieved 3- and 5-year OS rates of 83.0% and 74.0%, respectively. The favorable 5-year OS rate of 74.0% was not inferior to the results of the EORTC trial 40983 (51.2%) or other OS rates reported in clinical trials investigating the efficacy of NAC in patients with resectable CLM [10–12, 24–31]. Our findings confirmed the results of recent reviews and meta-analyses that demonstrated a lack of survival benefit for NAC in patients with clearly resectable lesions [24–31]. This implied that stratifying patient risk profiles is a key to maximizing the benefits of NAC in patients with resectable CLM.

We stratified eligible patients for preoperative chemotherapy by tumor characteristics that indicated unfavorable tumor biology, namely, four or more tumors, largest tumor diameter ≥ 5 cm, or concomitant resectable EHD. Recent data from several retrospective studies have shown that high-risk patients for disease recurrence received survival benefits from NAC [10–12], which suggests that patient stratification should be based on existing clinical risk scoring systems [13, 14]. However, the prognostic factors in these risk scoring systems were established at a time when effective cytotoxic agents were not available; thus, their utility in the era of modern chemotherapy is uncertain and should be reassessed to fit the current management of CLM. In this study, multivariate analysis of the CR-US group revealed that DFI from a primary disease of less than 12 months was the only predictive factor of impaired survival, which implies that CR-CLM patients with short DFI can be additional candidates for NAC. However, short DFI is the surrogate of refractory to adjuvant chemotherapy for primary disease. Hence, administration of NAC may not benefit CR-CLM patients with short DFI.

Favorable overall survival was achieved by upfront surgery in patients with CR-CLM and by NAC in patients with BR-CLM. However, the definition of borderline resectable disease needs to be refined over time in light of the following perspectives. First, the technical resectability of CLM may continue to evolve with advances in surgical techniques. Second, the optimal sites for resectable EHD should be identified, given that recent data have indicated that EHD sites considerably affect survival [32]. Finally, predictive biomarkers such as *RAS* or *BRAF* may help to identify CLM patients who will most benefit from NAC [33–36].

Although there were no differences in recurrence patterns after hepatectomy between the BR-NAC and BR-US groups, the resection rate for recurrences was higher in the BR-NAC group. Assessments of the recurrence patterns and resection rates for recurrent disease revealed that NAC for BR-CLM significantly reduced unresectable liver recurrences compared with patients that underwent upfront surgery for BR-CLM. Oba et al. demonstrated a unique clinical characteristic of colorectal cancer, in which the first relapse-related event does not reflect long-term survival, as second or third re-resections had curative potential for some relapse patients in their study [37]. Taking this into consideration, even though there were high recurrence rates in BR-CLM patients, decreasing unresectable recurrences by controlling the disease prior to surgery may have led to favorable OS rates in the BR-NAC group.

In this study, no patients in the BR-NAC group were contraindicated for surgery following NAC due to chemotherapy-associated liver damage. However, the incidence of major complications after hepatectomy was higher in the BR-NAC group than in the CR-US group. NAC has been reported to be associated with increased morbidity of subsequent liver surgery, depending on the intensity of the regime, number of cycles, and recovery period prior to surgery [16, 19, 20] In this study, we performed hepatectomy 4 weeks after oxaliplatin-based chemotherapy and 6 weeks after bevacizumab administration. Complications in the BR-NAC group were reversible, and there is a possibility that the higher rate of morbidity was associated with more extended and complicated procedures than that in CR-US group.

Several limitations of this study should be acknowledged. First, we compared survival rates of patients who received NAC for BR-CLM (BR-NAC group) with those of the CR-US group, who had lower risk profiles and underwent upfront surgery without NAC. Therefore, there were differences in patient backgrounds between the two groups that could have impacted the different outcomes. Indeed, the proportion of patients that received adjuvant chemotherapy after hepatectomy was higher in the BR-NAC group than in the CR-US group. However, the available evidence does not demonstrate a definitive advantage of adjuvant chemotherapy on OS in CLM patients [17]. Second, although we primarily administered oxaliplatin-based chemotherapy as NAC, the regimens were not uniform, especially in terms of targeted agents, which may have resulted in heterogeneous preoperative chemotherapy effects. Additional studies are needed to optimize NAC regimens.

## Conclusion

In conclusion, defining borderline resectable disease based on tumor characteristics optimizes CLM patient selection for preoperative chemotherapy. Favorable overall survival can be achieved by upfront surgery in patients with clearly resectable CLM and by NAC in patients with BR-CLM.

## Data Availability

The datasets used and/or analyzed during the current study are available from the corresponding author on reasonable request.
